# Clinical Outcome of Intra-Arterial Embolization for Treatment of Patients with Pelvic Trauma

**DOI:** 10.1155/2011/935484

**Published:** 2011-04-19

**Authors:** M. W. Barentsz, E. P. A. Vonken, J. A. van Herwaarden, L. P. H. Leenen, W. P. Th. M. Mali, M. A. A. J. van den Bosch

**Affiliations:** Department of Radiology, University Medical Center Utrecht, Room E.01.132, Heidelberglaan 100, 3584 CX Utrecht, The Netherlands

## Abstract

*Purpose*. To analyse the technical success of pelvic embolization in our institution and to assess periprocedural hemodynamic status and morbidity/mortality of all pelvic trauma patients who underwent pelvic embolization. *Methods*. A retrospective analysis of patients with a pelvic fracture due to trauma who underwent arterial embolization was performed. Clinical data, pelvic radiographs, contrast-enhanced CT-scans, and angiographic findings were reviewed. Subsequently, the technical success and peri-procedural hemodynamic status were evaluated and described. *Results*. 19 trauma patients with fractures of the pelvis underwent arterial embolization. Initially, 10/19 patients (53%) were hemodynamically unstable prior to embolization. Technical success of embolization was 100%. 14/19 patients (74%) were stable after embolization, and treatment success was high as 74%. *Conclusion*. Angiography with subsequent embolization should be performed in patients with a pelvic fracture due to trauma and hemodynamic instability, after surgical intervention or with a persistent arterial blush indicative of an active bleeding on CT.

## 1. Introduction

Trauma is one of the leading causes of death in patients under the age of 45 years [1]. Pelvic fractures occur in 4.0%–9.3% of patients with blunt trauma [2, 3]. These fractures should be considered severe, since mortality in these patients is high, ranging from 5.6% to 15.0% [2–8]. The mortality rate of hemodynamically unstable patient with pelvic fractures is even higher and ranges from 40% to 60% [5, 9–12]. Pelvic hemorrhage may originate from bone or vascular lesions. Venous bleeding occurs from the pelvic soft tissue, fracture ends, or the pelvic venous plexus. Arterial bleedings occur due to direct vascular laceration and are more frequently present in hemodynamically unstable patients [13, 14]. Management of hemodynamically unstable patients is aimed at controlling the venous and/or the arterial bleeding. Angiography and subsequent embolization can control arterial bleeding, while pelvic packing and external fixation encompass the current management of venous bleeding and bleeding from fracture sites. In the literature, specific indications for these treatment options remain controversial. 

**Figure 1:**
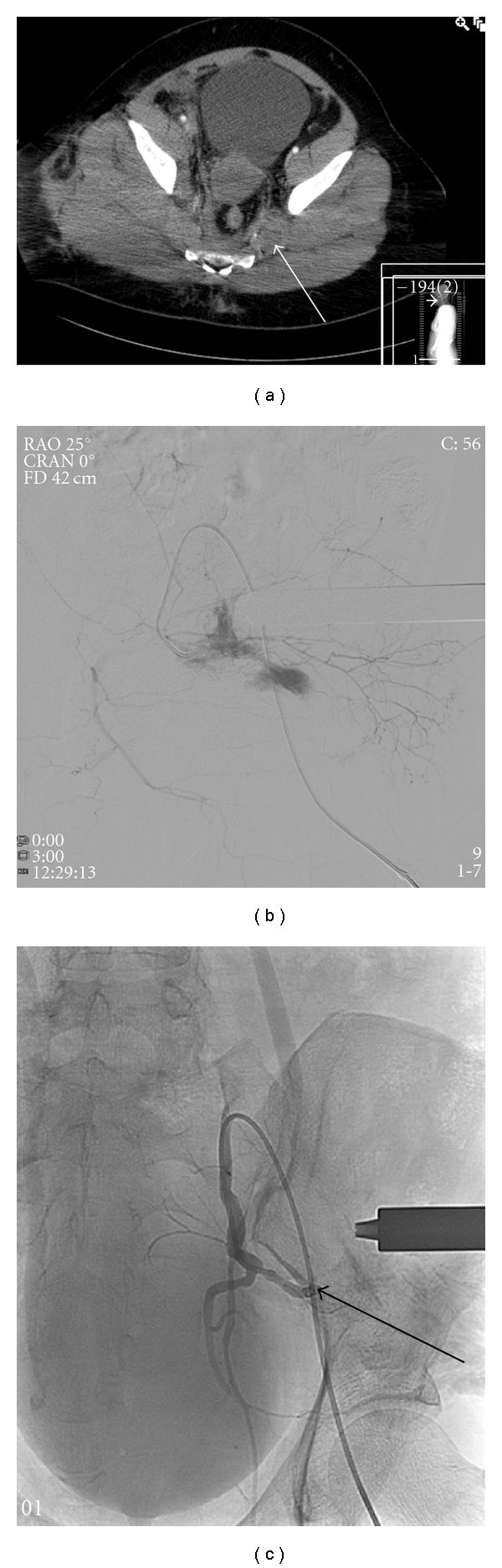
(a) Contrast-enhanced CT scan of a patient with a type C pelvic fracture after trauma. The arrow indicating a hematoma. (b) Selective catheterization of a branch from the left internal iliac artery showing a contrast blush, indicating an active bleeding. (c) Catheterization of the left internal iliac artery after coil embolization of the actively bleeding branch, with absence of any contrast blush (arrow indicating the coil in situ).

The presence of contrast extravasation on a contrast-enhanced computed tomography (CT) scan is a strong predictor of an arterial bleeding [15–18]. This can indicate injury to a specific artery in the pelvis region. With this localisation of injury provided by CT angiography, an interventional radiologist can actively search for the bleeding artery more efficiently. During the angiographic procedure, the abdominal aorta and its branches including the common iliac arteries, and internal iliac arteries (IIAs), are also visualized to identify macrovascular lesions. Extravasation of contrast material indicates an active bleeding. Other signs to look for during angiography are “missing” arteries, due to spasm and stagnation of contrast. Most frequently affected arteries include the superior gluteal, lateral sacral, iliolumbar, obturator, vesical, and inferior gluteal [19]. When an active bleeding site is found, the affected vessel is (super)selectively catheterized and subsequently embolized (Figure 1). The aim of the embolization is to immediately occlude the vessel with either coils or gelfoam, to reduce blood loss. In case of life-threatening hemodynamic instability and failure to find an arterial lesion, temporary nonselective embolization of both IIAs may be performed [20]. Success rates for arterial embolization for pelvic fractures are 80%–100% [19]. 

The aim of this study was to analyse the technical success of pelvic embolization and to assess peri-procedural hemodynamic status and morbidity/mortality of all pelvic trauma patients who underwent pelvic embolization in our institution in the last five years.

## 2. Materials and Methods

### 2.1. Patients

We retrospectively analyzed our database of patients who underwent pelvic embolization from January 2006 till October 2010 in our institution. All records of trauma patients were evaluated. Data examined included age, gender, cause of trauma, injuries, hemodynamic status at admission, pretreatment diagnostics, indication for intervention, localisation of bleeding, embolization procedure itself (i.e., arteries embolized, materials used, and duration of the intervention), and clinical outcome with hemodynamic status (within 30 days). Clinical data, pelvic radiographs, contrast-enhanced CT scans, and angiographic findings were compiled from the patient's medical, records, and the radiographic images were retrieved from the PACS system and reevaluated.

### 2.2. Technique

All patients were presented to the emergency department of our level I trauma center where Advanced Trauma Life Support (ATLS) protocols were followed. A whole-body CT examination (i.e., cerebral, cervical spine, chest, abdominal, and pelvic) was performed in all multiple trauma patients, if hemodynamic status permitted. An abdominal contrast-enhanced CT scan was performed with a total of 150 cc contrast, in arterial phase and delayed phase, to detect pelvic arterial lesions. Pelvic angiography was performed in two situations: when a contrast blush was identified on CT or when bleeding from pelvic fractures could not be easily controlled surgically and ongoing bleeding was probably present. The angiography was performed through a common femoral artery approach. A standard 5-French (F) pigtail was used with a 0.035 guidewire (Terumo, Leuven, Belgium). The catheter was placed in the distal aorta, just above the bifurcation, and angiography was performed in multiple views. Subsequently, the common iliac arteries and internal iliac arteries were visualized and selectively catheterized to identify active bleeding. If there was an angiographic evidence of arterial trauma, superselective catheterization of the feeding artery was performed with a 2.7 F microcatheter (Terumo, Leuven, Belgium), and embolization was performed with coils, ranging 2–6 millimetre in diameter (helical coils, nester coils 0.018 inches) or gelfoam (Spongostan). Subsequently, a final angiogram was performed to confirm absence of active bleeding. In combination with a decreasing need for blood transfusion, this is defined as technically successful embolization.

### 2.3. Follow Up

Follow up to evaluate treatment success of embolization was performed by chart review. Hemodynamic status, blood transfusion, and all (surgical) procedures, within 30 days after admission, were evaluated.

### 2.4. Definitions

As defined by other authors, technical success is achieved by successful embolization of the bleeding artery and the absence of extravasation of contrast after arterial embolization. 

Treatment success of embolization was achieved with stabilization of the hemodynamic status of the patient without the need of a surgical procedure for hemodynamic stabilisation (within 30 days). Hemodynamic stability was defined as no clinical signs of ongoing haemorrhage, and hemodynamic instability was defined as decreased ability of blood flow to meet the metabolic demands of the body [21]. The efficacy of arterial embolization was defined as the percentage of patients who had a technical successful embolization and treatment success with control of bleeding after embolization [20].

## 3. Results

During the study period, 19 trauma patients with fractures of the pelvis underwent arterial embolization. Six were female and 13 male, with a mean age of 45 years (range 15–76 y). Demographics and baseline characteristics are presented in Table 1. Mechanisms of injury included motor vehicle and motorcycle collisions (*n* = 8), vertical fall (*n* = 4), pedestrian or bike versus motor vehicle (*n* = 4), fall from stairs (*n* = 2), and crush (*n* = 1). Pelvic fractures were classified by the Tile and Arbeitsgemeinschaft Osteosynthese classification [22] in Type A (*n* = 3), Type B (*n* = 1), and Type C (*n* = 14), outlined in Table 2. One patient presented with an acetabulum fracture and was therefore not classified by the Tile classification. 8/19 (42%) patients were hemodynamically unstable at admission. In three hemodynamic unstable patients only an X-ray of the chest and pelvis was performed prior to angiography. In all other patients X-ray and a whole-body CT angiography (CTA) was performed. A contrast blush was detectable on the CTA scans of 11/15 (73%) patients. 

Indication for intervention was a decrease in haemoglobin (Hb) or an active bleeding (with or without a contrast blush on CT) in 10 patients (53%), a contrast blush on CT itself in 4 (21%), and hemodynamic instability in 5 (26%). Two patients were hemodynamic stable at admission; however, they became unstable and needed embolization consequently. During angiography, embolization was performed in all cases. Most frequently performed procedure was the embolization of a selective branch of the IIA. In four patients the IIA was embolized unilateral and in one patient the bilateral IIAs were embolized.  Table 3 provides an overview of all interventional procedures performed. Materials used for embolization were coils or gelfoam in most cases sometimes both were used. The duration of the embolization procedures ranged from 14 to 220 minutes, with a mean duration of 74 minutes (±49 minutes). 

Technical success of embolization was 100%. Initially, 10/19 (53%) patients were hemodynamic unstable prior to arterial embolization. After embolization 5/19 (26%) patients remained hemodynamic unstable. Consequently, 14/19 patients were stable after embolization, and treatment success was high as 74 percent. Three of the five hemodynamic unstable patients died due to hypovolemic shock (*n* = 2) and cerebral herniation (*n* = 1). The remaining two patients needed additional surgery for hemodynamic stabilization (i.e., decompression laparotomy and removal of pelvic packages).

## 4. Discussion

This study focuses on the technical and clinical success of embolization of trauma patients with a pelvic fracture. Angiography and embolization have evolved as a therapeutic modality in the treatment and stabilization of pelvic bleeding. The role of the interventional radiologist has increased in controlling bleeding in trauma patients. In our institution, we have used pelvic embolization, in addition to surgical packing and/or fixation with an increasing frequency over the past 5 years. 

In the current study, technical success rate (100%) is comparable to previously described results in the literature (95–100%) [4, 20, 23–26]. The overall mortality (15.8%) after embolization is also corresponding to the lower limits of to previously published results (13–47%) [4, 20, 23–27]. Treatment success, defined as stabilization of the hemodynamic status, without the need of a surgical procedure for hemodynamic stabilisation (<30 days) is slightly lower in our study (74%) than in other studies (81–95%) [20, 25, 26]. This could be the result of our definition, that is, an additional surgical procedure is necessary for stabilizing the hemodynamic status. When considering the clinically successful outcome (<30 days) of trauma patients after pelvic embolization including additional surgery, success rate is high as 84%.

The success rate is strongly related to the type of fracture. Anterior-posterior compression and vertical shear injuries (Tile classification Type A and C, resp.) are most likely to cause vascular injury. Lateral compression, in contrast, is least likely to disrupt the vascular bed [25]. Type C fractures are unstable by definition, and patients with this type of fracture, therefore, have a worse prognosis. The three mortalities in our study, all had a Type C fracture and were hemodynamic unstable at admission. Furthermore, two of these patients were too unstable for CT-scan at admission. These factors can be used as markers for prediction of outcome. Other predictors of death include posterior pelvic artery injury and need for fluid requirement to achieve hemodynamic stability [6]. 

Arterial embolization in patients with a pelvic fracture is a safe procedure. Morbidity attributed to the procedure itself was minimal. The patients who died in our study died due to the injuries sustained in the trauma (i.e., multiorgan failure, hypovolemic shock, and cerebral herniation). We did not encounter any serious complications like gluteal muscle necrosis, skin necrosis, and dissemination of an embolism as described in the literature [28–30]. It has been described that distal necrosis is more likely to occur when small sized particles are used. Since we used coils in combination with gelfoam, resulting in more proximal occlusion of the feeding artery, this complication did not occur in our patients. 

A multidisciplinary approach is essential for the management of hemodynamic unstable patients and controlling potentially lethal bleeding sites. Multidisciplinary management is particularly necessary with regards to the different treatment approaches of venous and arterial bleeding by the trauma surgeon and interventional radiologist, respectively. 

**Figure 2:**
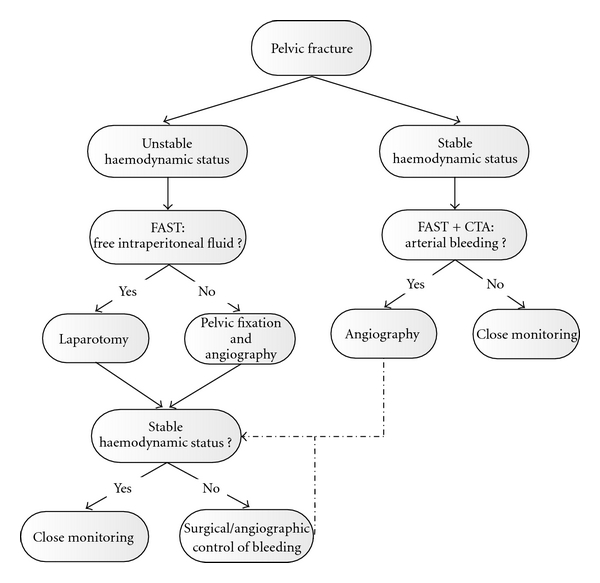
Treatment algorithm. FAST: focused assessment with sonography for trauma; CTA: computed tomography angiography.

In the management of patients with a pelvic fracture, the first important factor is the hemodynamic status. In case of hemodynamic instability, focused assessment with sonography for trauma (FAST) should be performed to detect intraperitoneal fluid. If present, the patient should be taken to the operation room for laparotomy. When the FAST shows no intraperitoneal fluid, the patient should be directed to the cath lab for angiography and embolization. In hemodynamically stable patients, first diagnostics are a FAST and contrast-enhanced CT scan. If a contrast blush is detected on the CT, angiography and arterial embolization should be performed. This treatment algorithm is provided in Figure 2. In haemodynamic unstable patients, in our institution, we do not prophylactically embolize both internal iliac arteries. We actively search for an active arterial bleeding, and if no contrast extravasation is detected, the hemorrhage may originate from bone or vascular lesions.

## 5. Conclusion

This study shows that angiography with subsequent embolization should be performed in patients with a pelvic fracture due to trauma and hemodynamic instability, after surgical intervention or with a persistent arterial blush on contrast-enhanced CT scan.

## Figures and Tables

**Table 1:** Baseline characteristics.

Characteristics	*N*	%
No. of patients	19	(100)
Age, median (yr)	45 ± 20.9	n.a.

Gender		
Female	6	(32)
Male	13	(68)

Hemodynamic status		
Stable	8	(42)
Unstable	11	(58)

Diagnostics		
X-ray	3	(16)
X-ray + CTA	16	(84)

Contrast blush on CTA		
Yes	11	(58)
No	4	(21)
n.a.	4	(21)

Pelvic fracture (Tile Classification)		
Type A	3	(16)
Type B	1	(5)
Type C	14	(74)
Acetabulum fracture only	1	(5)

n.a. not applicable; CTA: computed tomographic angiography.

**Table 2:** Tile classification of pelvic injuries [22].

Type A: Stable fractures (sacroiliac complex is intact)	
A1	Avulsion fractures
A2	Wing of ilium fractures
A3	Sacorcoccyx transverse fractures

Type B: partially stable fractures (partial disruption of the posterior sacroiliac complex)	

B1	Open book lesion
B2	Lateral compression lesion
B3	Bilateral compression lesion

Type C: unstable fractures (complete disruption of the posterior sacroiliac complex)	

C1	Unilateral lesion
C2	Bilateral lesion (one side B, one side C)
C3	Bilateral lesion (both sides C)

**Table 3:** Interventions.

	Indication for intervention	Localisation of bleeding	Material of embolization	Duration of embolization (min)
1	L kidney lesion + pelvic fracture with blush	inferior rectal artery + L renal artery	Coils	60
2	Decrease in Hb + blush	branches pudendal artery + EIA	Contour particles	60
3	Remaining blood loss	branches pudendal artery L + R	Coils	166
4	Active bleeding with blush	IIA + branches R hepatic	Gelfoam	61
5	Decrease in Hb	superior gluteal artery + iliolumbar artery	Gelfoam	14
6	Decrease in Hb	branch gluteal artery	Gelfoam	41
7	Decrease in Hb	branch IIA L + proximal IIA R	Coils	77
8	Decrease in Hb	pudendal artery L + R	Coils	82
9	Blush	branches IIA L	Coils	44
10	Hemodynamic instability	branches IIA R + branch IIA L	Coils + Histoacryl	220
11	Blush	branch IIA R	Gelfoam + coils	39
12	Blush	branch IIA R	Histoacryl	44
13	Active bleeding with blush	branch EIA L + gastric artery L	Gelfoam + coils	96
14	Gained hemodynamic instability + blush	branch IIA L (proximal + distal)	Coils	58
15	Gained hemodynamic instability	IIA L + R	Gelfoam + coils	26
16	Hemodynamic instable (no surgery possible) + blush	IIA L	Coils	90
17	Remaining blood loss	branches IIA L	Coils	106
18	Remaining hemodynamic instable + blush	branches IIA L + IIA R	Gelfoam + coils	71
19	Decrease in Hb	IIA L + pudendal artery R	Coils	43

IIA: Internal Iliac Artery; EIA: External Iliac Artery; L: left; R: right.
